# Supplementary material for: Advanced Microscopy for Liver and Gut Ultrastructural Pathology in Patients with MVID and PFIC Caused by MYO5B Mutations
Source: J Clin Med. 2021 Apr 28;10(9):1901. doi: 10.3390/jcm10091901 (PMC8125609; doi:10.3390/jcm10091901)
Supplement: Supplementary file 1 [file jcm-10-01901-s001.zip › Hess-Vogel-JCM-revised-Supplementary-Material.pdf]

Supplementary Figure S1. Ultrastructure and immuno-gold labelling from duodenal biopsies (bx #1) and enteroids (ent.) from MYO5B-PFIC patient #1and control duodenal biopsies (ctrl.). (a) Resin section of enterocytes from control biopsy. N=nucleus; scale bar= 2µm. (b) Anti-TGN46 label (arrows) at compartments in the vicinity of Golgi stacks in cryo-section of control biopsy; scale bar=500 nm. (c) Golgi stacks and associated TGN of normal morphology as seen in resin section of enterocyte from control biopsy; scale bar=500 nm. (d) Resin section of biopsy from patient #1 shows highly dilated compartments, likely TGN (marked by a cross: +) in close vicinity of poorly preserved Golgi stacks (arrows). Note swollen rER in the neighboring cell; scale bar=500 nm. (e) Dilated TGN in cryo-section of biopsy from patient #1 identified by respective label (arrows) at inflated cisternae and associated vesicles; scale bar=500 nm. (f) Golgi stack identification through giantin-label (arrows) in cryo-section of biopsy from patient #1; scale bar=1 µm. (g) Biopsy from patient #1 seen in cryo-section at low magnification with microvillus inclusions (asterisks), various huge autophagolysosomes and lysosomes (arrow-heads), as well inflated TGN (marked by cross: +); scale bar=5 µm. (h-l) Distribution of autophagic and lysosomal markers throughout the large catabolic organelles in biopsy and enteroid samples from patient #1. (h) Autophagic marker LC3 (arrows) and LAMP1 (arrow-heads) immuno-gold labeling; scale bar=500 nm. (j) Autophagic marker p62 labeling (arrows); scale bar=500 nm. (j-k) Cathepsin D label seen in biopsy #1 (j) versus enteroid (k); scale bars=500 nm. (l) LC3 label in enteroid; scale bar=500 nm.

Supplementary Figure S2. Cryo-fixed enteroids from MYO5B-PFIC patient #1. (a) Crypt-like area showing immature enterocytes with lysosomes (LY) and scattered electron-dense vesicles and tubules in the subapical cytoplasm, ranging in terms of size and shape between slim 60 nm-wide tubules (arrow-heads) and larger, more spherical organelles, likely aberrant recycling endosomes (RE: arrow); scale bar= 2 µm. (b) Slim, electron-dense tubular organelles (arrow-heads) occurring throughout the cytoplasm, but also laterally, likely representing normal RE. Arrow marks larger, presumably aberrant RE in the neighboring cell; scale bar=1 µm. (c) Pericentriolar apical region of middle-aged enterocyte showing numerous 60-100 nm-wide dense tubules (arrow-heads), considered as normal RE. Note medium-sized lysosomes (LY); scale bar=1 µm. (d) Mature enterocyte with large, aberrant RE (arrows) accumulating in the subapical cytoplasm; scale bar=2 µm. The patters of electron-dense tubular organelles documented here in panels (a-d) could be interpreted as smoothly graded series of transitional forms of RE, with the extremes of slim, normal RE (arrow-heads) on the one hand, and enlarged, aberrantly localizing MVID-specific RE (arrows) on the other hand. (e), (f) Vesicles attached to the apical plasma membrane, presumably preceding exocytosis; scale bars=100 nm. (g) Complex Golgi stacks and TGN with abundant associated vesicles indicate high metabolic activity of those compartments; scale bar=1 µm. (h) Autophagosome with intact contents, surrounded by 2 separate sheets of membrane bilayer; scale bar=500 nm. (i) Autophagolysosmes with quite undigested, partially well recognizable organelles

trapped within. Compartments limited by a single membrane bilayer; scale bar=500 nm. (j) Autophagolysosomes harboring partially degraded organelle remnants; scale bar=1 µm. (k) Large, terminal lysosomes; scale bar=1 µm,

(l) Enterocyte with denuded brush border; interdigitations of neighboring cells marked by double arrow-heads; scale bar=2 µm. (m) Pathognomonic microvillus inclusion (asterisk); scale bar=1 µm.

Supplementary Figure S3. Cryo-fixed enteroids from control biopsy. (a) Overview micrograph showing normal morphology of lysosomes (LY), mitochondria (M), Golgi stack (G) and putative recycling endosomes (arrow-heads); scale bar=1 µm. (b) Detail view highlighting electron-dense, slim tubules (arrow-heads) representing likely normal recycling endosomes; scale bar=500 nm.

Supplementary Figure S4. Apical marker immuno-gold EM of gut and enteroid samples from patient #1, immunoblot validation of #596 anti-CFTR mAB, and resin sections of MVID features seen in biopsy of patient #2. (a) Syntaxin3 label at aberrant, subapical RE of enteroid from patient #1; scale bar=500 nm. (b) Immuno-blot of HeLa cells to show specificity of anti-CFTR mAb #596. HeLa cells were stably transfected with CFTR wild type or CFTR-F508Δ mutant constructs and treated with 20 µM protease inhibitor ALLN or proteasome inhibitor MG132 for 24 hrs. as indicated. The upper from two CFTR bands represents the mature, fully glycosylated CFTR form. Anti-β-tubulin staining was used for the normalization of protein amounts analyzed by the Western blot. (c) Normal brush border localization of CFTR in control biopsy versus distinct mislocalization (arrows) in biopsy (d) and, to a minor extent, in enteroid sample (e) from patient #1; scale bars=500 nm. (f) Normal brush border localization of DPP4 in control biopsy versus congruent mislocalization (arrows) in biopsy (g) and enteroid sample (h) from patient #1; scale bars=500 nm. (i) Aldehyde-fixed duodenal biopsy of patient #2 shows clearly PAS-positive, but poorly preserved organelles in the enterocytes' periphery after standard preparation (protocol I). LY marks enlarged lysosome; scale bar=1 µm. (j-k) Additional stabilization of the aldehyde-fixed biopsy from patient #2 through HPF/FS (protocol IV) considerably improves ultrastructural preservation of aberrant subapical RE (j), including respective PAS-reaction of the organelles (k); scale bars=1 µm.

Supplementary Figure S5. Immuno-gold EM of cryo-sections from formaldehyde-fixed liver biopsy from MYO5B-PFIC patient #1. (a) Rab11a immuno-gold particles (VU57) are partially located at ≈120 nm-wide vesicles (arrows), but not at vesicles positive for ER-marker PDI (arrow-heads) in the hepatocytes apical region facing the bile canaliculus (BC); scale bar=200 nm. (b) Anti-giantin (arrows) identifies "swollen membrane sacks" as Golgi stacks/TGN, weak, scattered anti-PDI label marked by arrow-heads; scale bar=500 nm.

Supplementary Figure S1. Ultrastructure and immuno-gold labelling from duodenal biopsies (bx #1) and enteroids (ent.) from MYO5B-PFIC patient #1and control duodenal biopsies (ctrl.). (a) Resin section of enterocytes from control biopsy. N=nucleus; scale bar= 2µm. (b) Anti-TGN46 label (arrows) at compartments in the vicinity of Golgi stacks in cryo-section of control biopsy; scale bar=500 nm. (c) Golgi stacks and associated TGN of normal morphology as seen in resin section of enterocyte from control biopsy; scale bar=500 nm. (d) Resin section of biopsy from patient #1 shows highly dilated compartments, likely TGN (marked by a cross: +) in close vicinity of poorly preserved Golgi stacks (arrows). Note swollen rER in the neighboring cell; scale bar=500 nm. (e) Dilated TGN in cryo-section of biopsy from patient #1 identified by respective label (arrows) at inflated cisternae and associated vesicles; scale bar=500 nm. (f) Golgi stack identification through giantin-label (arrows) in cryo-section of biopsy from patient #1; scale bar=1 µm. (g) Biopsy from patient #1 seen in cryo-section at low magnification with microvillus inclusions (asterisks), various huge autophagolysosomes and lysosomes (arrow-heads), as well inflated TGN (marked by cross: +); scale bar=5 µm. (h-l) Distribution of autophagic and lysosomal markers throughout the large catabolic organelles in biopsy and enteroid samples from patient #1. (h) Autophagic marker LC3 (arrows) and LAMP1 (arrow-heads) immuno-gold labeling; scale bar=500 nm. (j) Autophagic marker p62 labeling (arrows); scale bar=500 nm. (j-k) Cathepsin D label seen in biopsy #1 (j) versus enteroid (k); scale bars=500 nm. (l) LC3 label in enteroid; scale bar=500 nm.

Supplementary Figure S2. Cryo-fixed enteroids from MYO5B-PFIC patient #1. (a) Crypt-like area showing immature enterocytes with lysosomes (LY) and electron-dense recycling endosomes (RE: arrow-heads) of apparently normal morphology. Note just few, scattered apical vesicles but absence of aberrant RE in the subapical region; scale bar= 2 µm. (b) Slim, electron-dense tubular organelles (arrow-heads) occurring throughout the cytoplasm, but also laterally, likely representing normal RE. Arrow marks larger, presumably aberrant RE in the neighboring cell; scale bar=1 µm. (c) Pericentriolar apical region of middle-aged enterocyte showing numerous 60-100 nm-wide dense tubules (arrow-heads), considered as normal RE. Note medium-sized lysosomes (LY); scale bar=1 µm. (d) Mature enterocyte with large, aberrant RE (arrows) accumulating in the subapical cytoplasm; scale bar=2 µm. The patters of electron-dense tubular organelles documented here in panels (a-d) could be interpreted as smoothly graded series of transitional forms of RE, with the extremes of slim, normal RE (arrow-heads) on the one hand, and enlarged, aberrantly localizing MVID-specific RE (arrows) on the other hand. (e), (f) Vesicles attached to the apical plasma membrane, presumably preceding exocytosis; scale bars=100 nm. (g) Complex Golgi stacks and TGN with abundant associated vesicles indicate high metabolic activity of those compartments; scale bar=1 µm. (h) Autophagosome with intact contents, surrounded by 2 separate sheets of membrane bilayer; scale bar=500 nm. (i) Autophagolysosmes with quite undigested, partially well recognizable organelles trapped within. Compartments limited

by a single membrane bilayer; scale bar=500 nm. (j) Autophagolysosomes harboring partially degraded organelle remnants; scale bar=1 µm. (k) Large, terminal lysosomes; scale bar=1 µm, (l) Enterocyte with denuded brush border; interdigitations of neighboring cells marked by double arrow-heads; scale bar=2 µm. (m) Pathognomonic microvillus inclusion (asterisk); scale bar=1 µm.

Supplementary Figure S3. Cryo-fixed enteroids from control biopsy. (a) Overview micrograph showing normal morphology of lysosomes (LY), mitochondria (M), Golgi stack (G) and putative recycling endosomes (arrow-heads); scale bar=1 µm. (b) Detail view highlighting electron-dense, slim tubules (arrow-heads) representing likely normal recycling endosomes; scale bar=500 nm.

Supplementary Figure S4. Apical marker immuno-gold EM of gut and enteroid samples from patient #1, immunoblot validation of #596 anti-CFTR mAB, and resin sections of MVID features seen in biopsy of patient #2. (a) Syntaxin3 label at aberrant, subapical RE of enteroid from patient #1; scale bar=500 nm. (b) Immuno-blot of HeLa cells to show specificity of anti-CFTR mAb #596. HeLa cells were stably transfected with CFTR wild type or CFTR-F508Δ mutant constructs and treated with 20 µM protease inhibitor ALLN or proteasome inhibitor MG132 for 24 hrs. as indicated. The upper from two CFTR bands represents the mature, fully glycosylated CFTR form. Anti-β-tubulin staining was used for the normalization of protein amounts analyzed by the Western blot. (c) Normal brush border localization of CFTR in control biopsy versus distinct mislocalization (arrows) in biopsy (d) and, to a minor extent, in enteroid sample (e) from patient #1; scale bars=500 nm. (f) Normal brush border localization of DPP4 in control biopsy versus congruent mislocalization (arrows) in biopsy (g) and enteroid sample (h) from patient #1; scale bars=500 nm. (i) Aldehyde-fixed duodenal biopsy of patient #2 shows clearly PAS-positive, but poorly preserved organelles in the enterocytes' periphery after standard preparation (protocol I). LY marks enlarged lysosome; scale bar=1 µm. (j-k) Additional stabilization of the aldehyde-fixed biopsy from patient #2 through HPF/FS (protocol IV) considerably improves ultrastructural preservation of aberrant subapical RE (j), including respective PAS-reaction of the organelles (k); scale bars=1 µm.

Supplementary Figure S5. Immuno-gold EM of cryo-sections from formaldehyde-fixed liver biopsy from MYO5B-PFIC patient #1. (a) Rab11a immuno-gold particles (VU57) are partially located at ≈120 nm-wide vesicles (arrows), but not at vesicles positive for ER-marker PDI (arrow-heads) in the hepatocytes apical region facing the bile canaliculus (BC); scale bar=200 nm. (b) Anti-giantin (arrows) identifies "swollen membrane sacks" as Golgi stacks/TGN, weak, scattered anti-PDI label marked by arrow-heads; scale bar=500 nm.

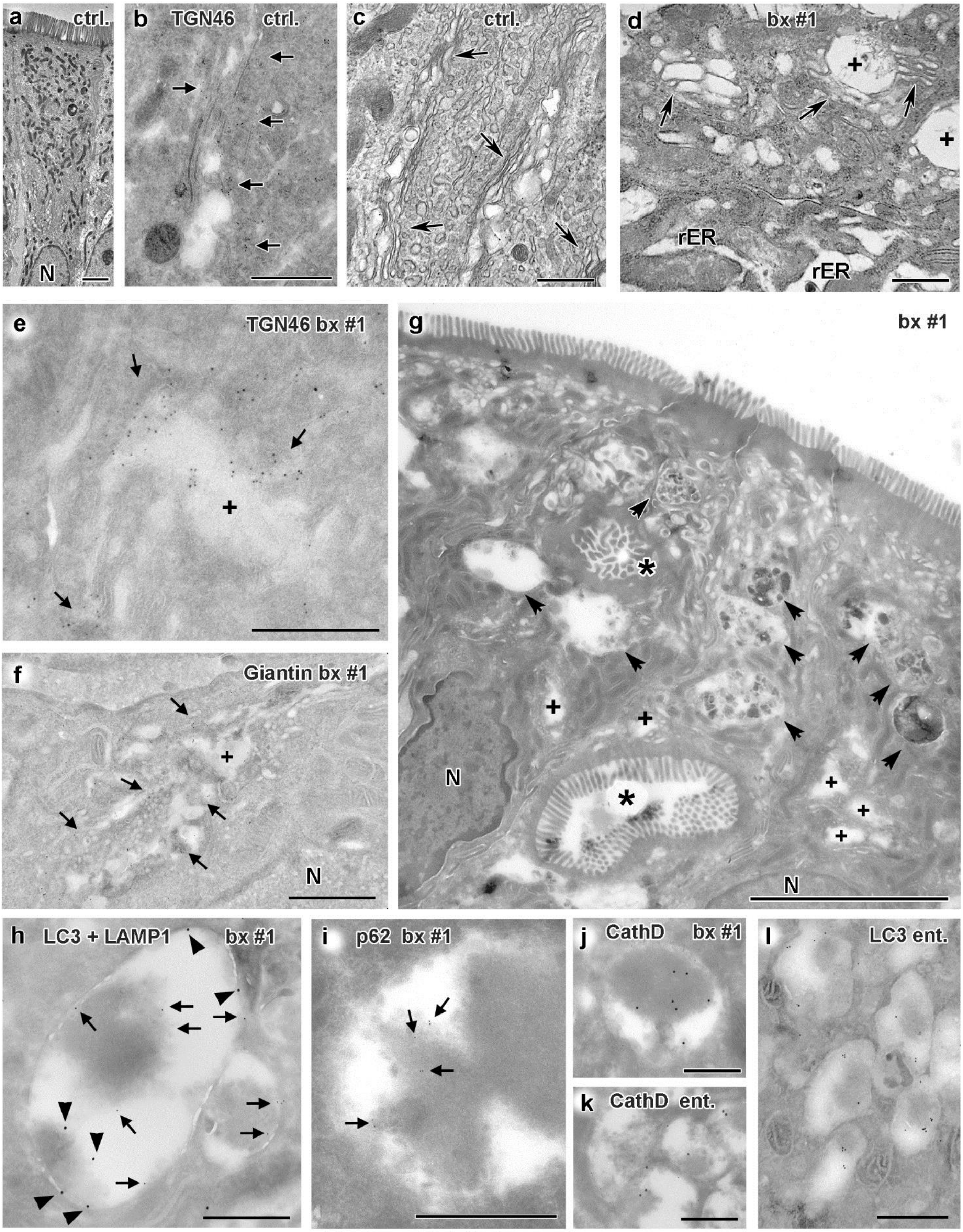
a ctrl.
N
b TGN46 ctrl.
c ctrl.
d bx #1
rER
rER
e TGN46 bx #1
f Giantin bx #1
N
g bx #1
N
N
h LC3 + LAMP1 bx #1
i p62 bx #1
j CathD bx #1
k CathD ent.
l LC3 ent.

Figure S1

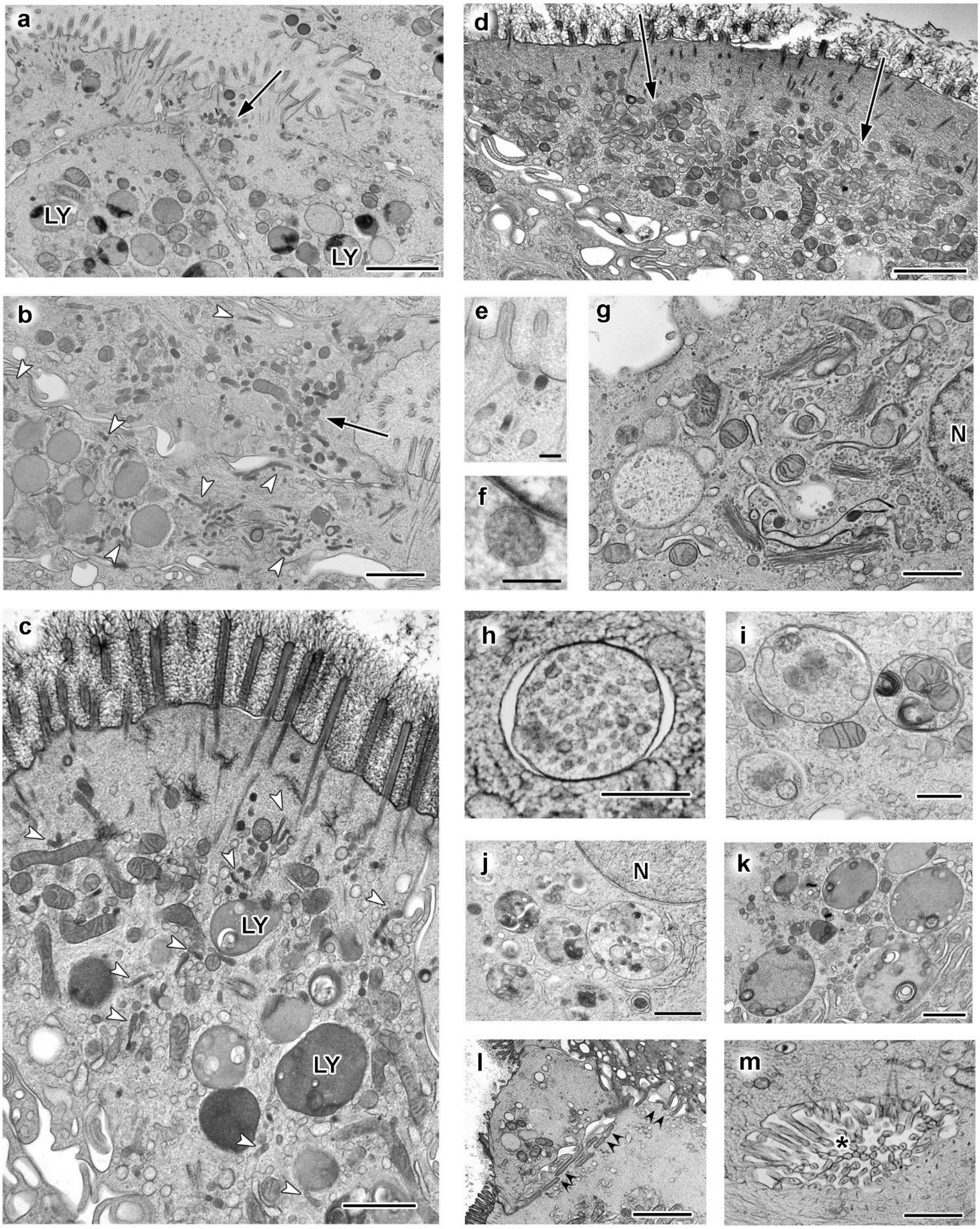

Figure S2

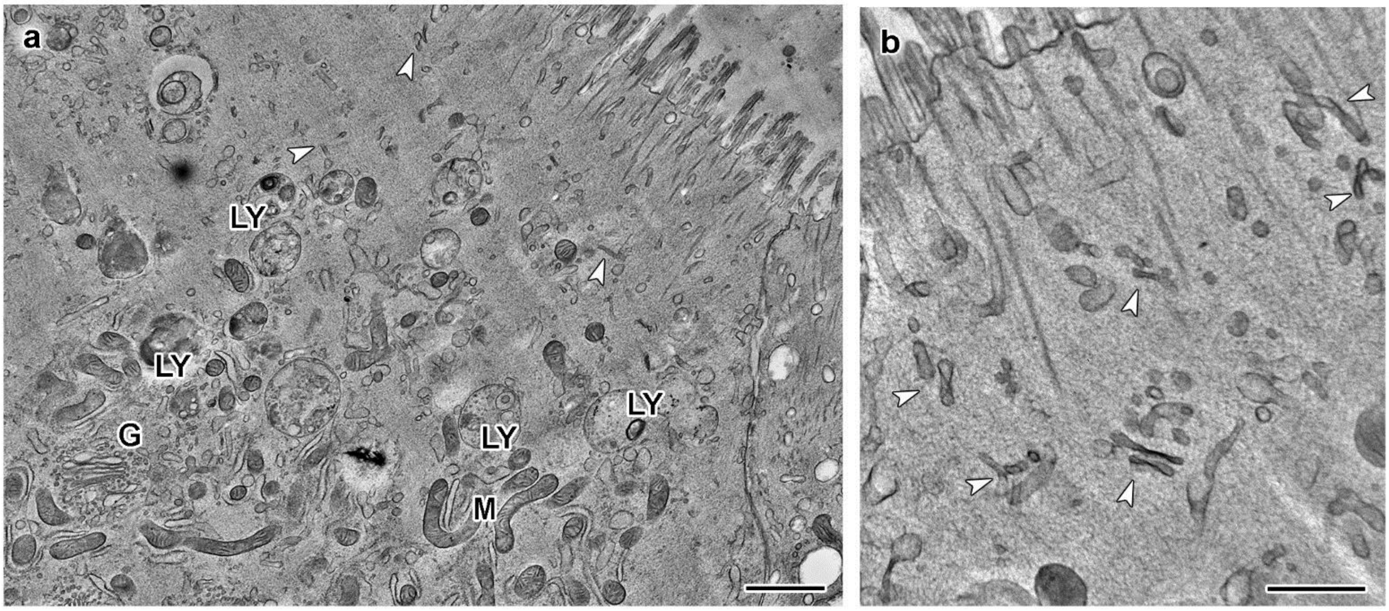

Figure S3

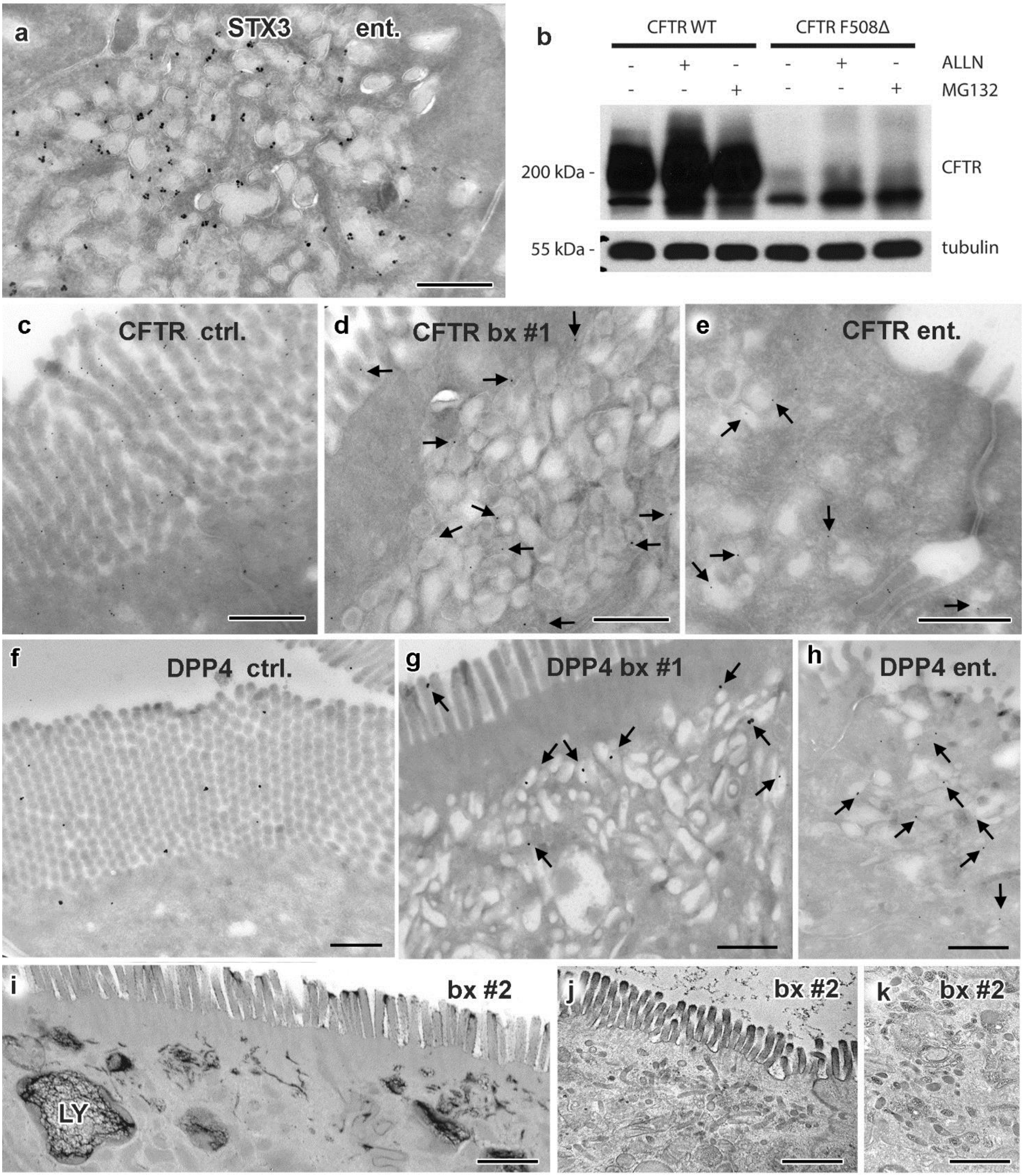
a
STX3
ent.
b
CFTR WT
CFTR F508Δ
ALLN
MG132
- + - - + -
- - + - - +
200 kDa -
55 kDa -
CFTR
tubulin
c
CFTR ctrl.
d
CFTR bx #1
e
CFTR ent.
f
DPP4 ctrl.
g
DPP4 bx #1
h
DPP4 ent.
i
bx #2
LY
j
bx #2
k
bx #2

Figure S4

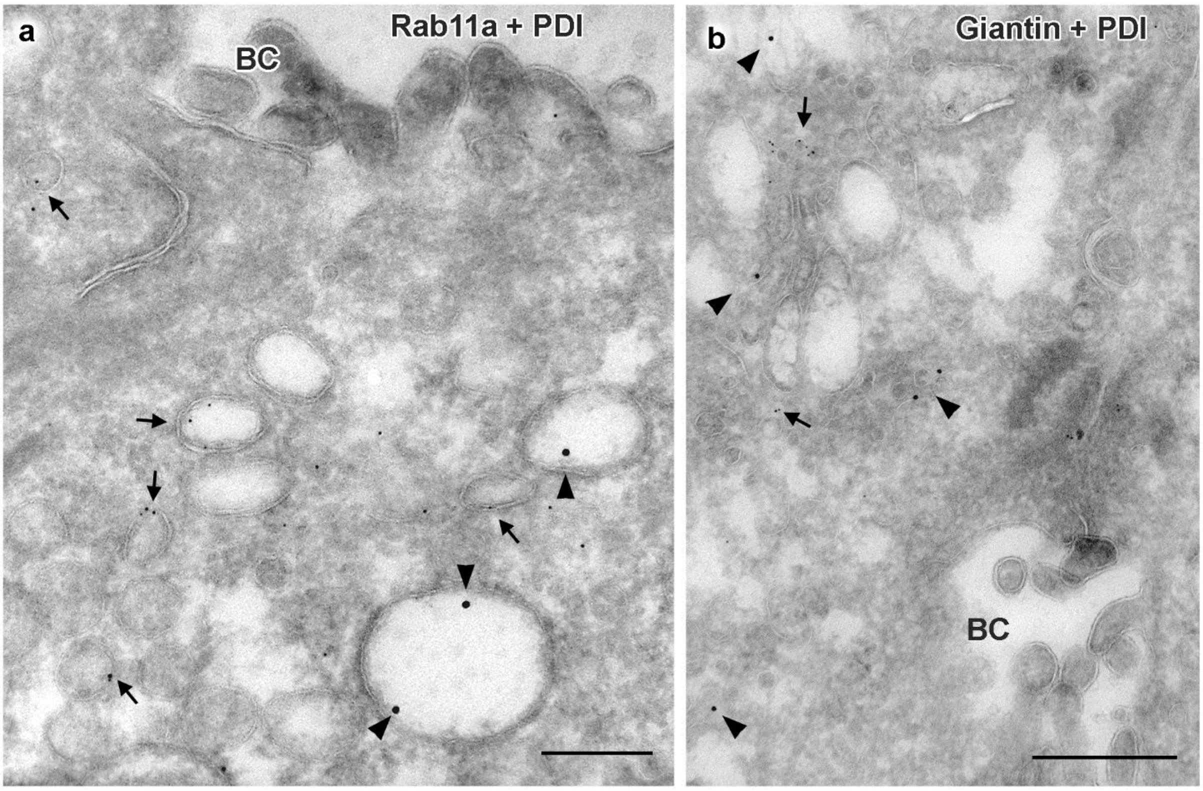

Figure S5

Supplementary Table 1. Immuno-labeling patterns in intestinal and liver biopsies from MYO5B-PFIC patient #1, observed by fluorescence microscopy (IF) and EM

	Protein	Duodenum normal	Duodenum abnormal	Notes	Liver normal	Liver abnormal
IF	syntaxin3		X		*not done*	
IF	NHE3		X		*not done*	
IF	Rab11		X			X
IF	myo5b	X	X		X	X
IF	DPP4	X			X	
IF	BSEP	*not done*				X
IF	MRP2	*not done*				X
EM	Rab11		X	in / at aberrant, subapical recycling endosomes		X
EM	Rab11a		X			X
EM	Rab8a		X		X	
EM	DPP4		X		X	
EM	NHE3		X		*not done*	
EM	syntaxin3		X		*not done*	
EM	CFTR	X	X		*not done*	
EM	PDI	X		in / at enlarged organelles	X	
EM	giantin	X			X	
EM	TGN46	X			X	
EM	M6PR	X			*not done*	
EM	LC3	X			*not done*	
EM	p62/SQSTM1	X			*not done*	
EM	LAMP1	X			*not done*	
EM	cathepsin D	X			*not done*	
EM	BSEP	*not done*			X	X
EM	MRP2	*not done*			X	

Supplementary methods. Advantages and disadvantages of the specimen preparation protocols employed

Protocol I: Chemical fixation and standard processing for ultrastructural analyses of biopsies.

Pro: Aldehyde fixation followed by post-fixation and -staining with heavy metals, dehydration at RT/+4°C and final epoxy resin embedding (1) is a well established, inexpensive routine method without need for special infrastructure.

Con: Ultrastructural preservation is acceptable. However, reliability is limited because of artifactual ultrastructure alterations induced by the relatively slow interaction of toxic fixatives with live material (2), and other adverse properties of reagents used for post-staining and dehydration at RT/+4°C (3).

Protocol II: Cryo-fixation for ultrastructural analyses of organoids

Pro: High-pressure freezing (HPF) of live specimens (4, 5), immobilizes the dynamic subcellular architecture within less than 50 milliseconds (6, 7) circumventing the problem of artifactual (membrane) deformations (2) that are inevitably accompany fixation through chemicals (3). The frozen samples are subsequently exposed to fixative-containing organic solvents at very low temperatures of about –90°C (i.e., processed by freeze-substitution (FS): Ref. (8)). This way, the cryo-immobilized samples become dehydrated, chemically stabilized and stained in a very slow, gentle and controlled manner before they are normally embedded in epoxides.

Con: Expensive instrumentation and respective operating skills are mandatory. Because of the complex logistic requirements this special technique is usually not applied to fresh patient biopsies (3).

Protocol III: Chemical fixation for cryo-ultramicrotomy and immuno-EM of biopsies and organoids (Tokoyasu-technique (9))

Pro: Aldehyde-fixed samples, subsequently cryo-protected with a concentrated sucrose solution are cut at about −110°C in a cryo-ultramicrotome to ≈100nm-thin sections that are thawed and immuno-labeled with (gold-conjugated) antibodies. Organic solvents, including embedding resins which may reduce antigenicity or mask relevant epitopes are avoided; thus, comparatively high labeling efficiency is achieved (10).

Con: Chemical fixation always induces certain artifacts. In addition, expensive instrumentation and respective handling skills are required.

Protocol IV: Chemical fixation combined with HPF and FS for ultrastructural analyses of biopsies.

Pro: This "hybrid technique" preserves certain fragile endomembranes better than standard chemical fixation, post-fixation and dehydration at RT/+4°C (11, 12). It is recommended when direct rapid freezing of live samples (protocol II) is hardly possible (e.g., with patient biopsies or infectious material). Aldehyde-fixed samples are conveniently shipped to collaborators equipped for cryo-fixation.

Con: This approach is inferior to direct cryo-fixation of live samples because of inevitable fixation artifacts.

Workflow of specimen preparation and cytochemistry

**Protocol I**	**Chemical fixation and resin embedding**	(4 % FA [pH 7.2; 1 to >3d] →) 2.5% GA → 0.5% OsO_4 → 0.2% UA → EtOH → acetone → epoxide
**Protocol II**	**Cryo-fixation and resin embedding**	HPF → FS [acetone + 0.5% OsO_4 + 0.08% UA + 4.2% H_2O] → acetone → epoxide
**Protocol III**	**Chemical fixation and cryo-ultramicrotomy for Tokuyasu-immuno-EM (9)**	4 % FA [pH 7.2; 30 min to >3d] → 4% FA [pH 11.0; 30 min] → 2.3M sucrose → cryo-ultramicrotomy at −110°C → PBS at RT → immuno-gold → 2% UA → 0.4% UA → methylcellulose
**Protocol IV**	**Chemical fixation supplemented by cryo-fixation for resin embedding**	4% FA → HPF → FS → acetone → epoxide
**PAS-correlate for EM**	**Thiéry-technique (13)**	PA → TCH → SP [at +50°C]

1. Hayat MA. *Principles and techniques of electron microscopy. Biological applications.* Cambridge: Cambridge University Press; 2000.
2. Murk JL, Posthuma G, Koster AJ, Geuze HJ, Verkleij AJ, Kleijmeer MJ, et al. Influence of aldehyde fixation on the morphology of endosomes and lysosomes: quantitative analysis and electron tomography. *J Microsc.* 2003;212(Pt 1):81-90.
3. Pfeiffer S, Vielhaber G, Vietzke JP, Wittern KP, Hintze U, and Wepf R. High-pressure freezing provides new information on human epidermis: simultaneous protein antigen and lamellar lipid structure preservation. Study on human epidermis by cryoimmobilization. *J Invest Dermatol.* 2000;114(5):1030-8.
4. Müller M, and Moor H. Cryofixation of thick specimens by high-pressure freezing. In: Revel JP, Barnard T, and Haggins GH eds. *The science of biological specimen preparation*. Chicago: SEM Inc. AMF O'Hare; 1984:131-8.
5. Studer D, Michel M, and Muller M. High pressure freezing comes of age. *Scanning Microsc Suppl.* 1989;3:253-68; discussion 68-69.
6. Moor H. Theory and practice of high pressure freezing. In: Steinbrecht RA, and Zierold K eds. *Cryotechniques in biological electron microscopy*. Berlin, Heidelberg: Springer; 1987:175-91.
7. Studer D, Michel M, Wohlwend M, Hunziker EB, and Buschmann MD. Vitrification of articular cartilage by high-pressure freezing. *J Microsc.* 1995;179 (Pt 3):321-32.
8. Humbel B, and Müller M. Freeze substitution and low temperature embedding. In: Müller M, Becker RP, and Wolosewick JJ eds. *The science of biological specimen preparation 1985*. Chicago: SEM Inc. AMF O'Hare; 1986:175-83.
9. Tokuyasu KT. A technique for ultracryotomy of cell suspensions and tissues. *The Journal of cell biology.* 1973;57(2):551-65.
10. Griffiths G. *Fine structure immunocytochemistry.* Berlin: Springer; 1993.
11. Sosinsky GE, Crum J, Jones YZ, Lanman J, Smarr B, Terada M, et al. The combination of chemical fixation procedures with high pressure freezing and freeze substitution preserves highly labile tissue ultrastructure for electron tomography applications. *Journal of structural biology.* 2008;161(3):359-71.
12. Mobius W. Cryopreparation of biological specimens for immunoelectron microscopy. *Ann Anat.* 2009;191(3):231-47.
13. Thiéry JP. Mise en évidence des polysaccharides sur coupes fines en microscopie électronique. *J Microscopie (Paris).* 1967;6:987-1018.

Supplementary methods. Advantages and disadvantages of the specimen preparation protocols employed

Protocol I: Chemical fixation and standard processing for ultrastructural analyses of biopsies.

Pro: Aldehyde fixation followed by post-fixation and -staining with heavy metals, dehydration at RT/+4°C and final epoxy resin embedding (1, 2) is a well established, inexpensive routine method without need for special infrastructure.

Con: Ultrastructural preservation is acceptable. However, reliability is limited because of artifactual ultrastructure alterations induced by the relatively slow interaction of toxic fixatives with live material (3), and other adverse properties of reagents used for post-staining and dehydration at RT/+4°C (4).

Protocol II: Cryo-fixation for ultrastructural analyses of organoids

Pro: High-pressure freezing (HPF) of live specimens (5, 6), immobilizes the dynamic subcellular architecture within less than 50 milliseconds (7, 8) circumventing the problem of artifactual (membrane) deformations (3) that are inevitably accompany fixation through chemicals (4). The frozen samples are subsequently exposed to fixative-containing organic solvents at very low temperatures of about –90°C (i.e., processed by freeze-substitution (FS): Ref. (9)). This way, the cryo-immobilized samples become dehydrated, chemically stabilized and stained in a very slow, gentle and controlled manner before they are normally embedded in epoxides.

Con: Expensive instrumentation and respective operating skills are mandatory. Because of the complex logistic requirements this special technique is usually not applied to fresh patient biopsies (4).

Protocol III: Chemical fixation for cryo-ultramicrotomy and immuno-EM of biopsies and organoids (Tokoyasu-technique (10))

Pro: Aldehyde-fixed samples, subsequently cryo-protected with a concentrated sucrose solution are cut at about −110°C in a cryo-ultramicrotome to ≈100nm-thin sections that are thawed and immuno-labeled with (gold-conjugated) antibodies. Organic solvents, including embedding resins which may reduce antigenicity or mask relevant epitopes are avoided; thus, comparatively high labeling efficiency is achieved (11).

Con: Chemical fixation always induces certain artifacts. In addition, expensive instrumentation and respective handling skills are required.

Protocol IV: Chemical fixation combined with HPF and FS for ultrastructural analyses of biopsies.

Pro: This "hybrid technique" preserves certain fragile endomembranes better than standard chemical fixation, post-fixation and dehydration at RT/+4°C (12, 13). It is recommended when direct rapid freezing of live samples (protocol II) is hardly possible (e.g., with patient biopsies or infectious material). Aldehyde-fixed samples are conveniently shipped to collaborators equipped for cryo-fixation.

Con: This approach is inferior to direct cryo-fixation of live samples because of inevitable fixation artifacts.

Workflow of specimen preparation and cytochemistry

**Protocol I**	**Chemical fixation and resin embedding**	(4 % FA [pH 7.2; 1 to >3d] →) 2.5% GA → 0.5% OsO4 → 0.2% UA → EtOH → acetone → epoxide
**Protocol II**	**Cryo-fixation and resin embedding**	HPF → FS [acetone + 0.5% OsO4 + 0.08% UA + 4.2% H2O] → acetone → epoxide
**Protocol III**	**Chemical fixation and cryo-ultramicrotomy for Tokuyasu-immuno-EM (10)**	4 % FA [pH 7.2; 30 min to >3d] → 4% FA [pH 11.0; 30 min] → 2.3M sucrose → cryo-ultramicrotomy at −110°C → PBS at RT → immuno-gold → 2% UA → 0.4% UA → methylcellulose
**Protocol IV**	**Chemical fixation supplemented by cryo-fixation for resin embedding**	4% FA → HPF → FS → acetone → epoxide
**PAS-correlate for EM**	**Thiéry-technique (14)**	PA → TCH → SP [at +50°C]

1. Hayat MA. *Principles and techniques of electron microscopy. Biological applications.* Cambridge: Cambridge University Press; 2000.
2. Graham L, and Orenstein JM. Processing tissue and cells for transmission electron microscopy in diagnostic pathology and research. *Nature protocols.* 2007;2(10):2439-50.
3. Murk JL, Posthuma G, Koster AJ, Geuze HJ, Verkleij AJ, Kleijmeer MJ, et al. Influence of aldehyde fixation on the morphology of endosomes and lysosomes: quantitative analysis and electron tomography. *J Microsc.* 2003;212(Pt 1):81-90.
4. Pfeiffer S, Vielhaber G, Vietzke JP, Wittern KP, Hintze U, and Wepf R. High-pressure freezing provides new information on human epidermis: simultaneous protein antigen and lamellar lipid structure preservation. Study on human epidermis by cryoimmobilization. *J Invest Dermatol.* 2000;114(5):1030-8.
5. Müller M, and Moor H. Cryofixation of thick specimens by high-pressure freezing. In: Revel JP, Barnard T, and Haggins GH eds. *The science of biological specimen preparation*. Chicago: SEM Inc. AMF O'Hare; 1984:131-8.
6. Studer D, Michel M, and Muller M. High pressure freezing comes of age. *Scanning Microsc Suppl.* 1989;3:253-68; discussion 68-69.
7. Moor H. Theory and practice of high-pressure freezing. In: Steinbrecht RA, and Zierold K eds. *Cryotechniques in biological electron microscopy*. Berlin, Heidelberg: Springer; 1987:175-91.
8. Studer D, Michel M, Wohlwend M, Hunziker EB, and Buschmann MD. Vitrification of articular cartilage by high-pressure freezing. *J Microsc.* 1995;179 (Pt 3):321-32.
9. Humbel B, and Müller M. Freeze substituion and low temperature embedding. In: Müller M, Becker RP, and Wolosewick JJ eds. *The science of biological specimen preparation 1985*. Chicago: SEM Inc. AMF O'Hare; 1986:175-83.
10. Tokuyasu KT. A technique for ultracryotomy of cell suspensions and tissues. *The Journal of cell biology.* 1973;57(2):551-65.
11. Griffiths G. *Fine structure immunocytochemistry.* Berlin: Springer; 1993.
12. Sosinsky GE, Crum J, Jones YZ, Lanman J, Smarr B, Terada M, et al. The combination of chemical fixation procedures with high pressure freezing and freeze substitution preserves highly labile tissue ultrastructure for electron tomography applications. *Journal of structural biology.* 2008;161(3):359-71.
13. Mobius W. Cryopreparation of biological specimens for immunoelectron microscopy. *Ann Anat.* 2009;191(3):231-47.
14. Thiéry JP. Mise en évidence des polysaccharides sur coupes fines en microscopie électronique. *J Microscopie (Paris).* 1967;6:987-1018.
